# Primary stabbing headache in a tertiary headache centre

**DOI:** 10.1186/s10194-025-02155-4

**Published:** 2025-10-16

**Authors:** Simone Braca, Amy Gimson, Peter J. Goadsby

**Affiliations:** 1 NIHR King’s Clinical Research Facility, and SLaM Biomedical Research Facility, and Wolfson Sensory, Pain and Regeneration Centre, King’s College London, London, UK; 2 King Abdullah Universiy of Science and Technology, Thuwal, Saudi Arabia; 3 Wellcome Foundation Building, King’s College Hospital, London, SE5 9PJ UK

## Abstract

**Introduction:**

Primary stabbing headache (PSH) is a short-lasting head pain occurring spontaneously in the absence of underlying structural causes. Although it is a frequent disorder, with a reported lifetime prevalence of 35.2% in the general population, its pathophysiological underpinnings remain incompletely understood. Notably, PSH frequently coexists with other primary headache disorders, particularly migraine, suggesting a possible shared biology.

**Methods:**

We retrospectively reviewed the medical records as a service evaluation of all patients diagnosed with PSH, using the International Classification of Headache Disorders, third edition, in our clinic between 2018 and 2025 as a service evaluation. Data on demographic variables and the presence of other primary headache disorders were collected and analysed. Concurrently, we conducted a systematic literature search according to PRISMA guidelines. A comprehensive search of PubMed/MEDLINE and EMBASE via Scopus through April 2025 identified studies examining the relationship between PSH and other primary headache disorders.

**Results:**

The retrospective analysis identified 68 patients who met the strict diagnostic criteria for PSH. Notably, 90% of these individuals were found to have a concomitant migraine diagnosis, while hemicrania continua (HC) and cluster headache (CH) were reported in 7% and 10% of cases, respectively. From the literature search, 27 eligible studies were included. Overall, a significant rate of co-occurrence between PSH and migraine was consistently reported, sometimes exceeding two-thirds of PSH cases. Likewise, in migraine cohorts PSH has been repeatedly observed at substantial rates, suggesting a close link between the two conditions. PSH was commonly reported within trigeminal autonomic cephalalgias (TACs), occurring in 36–41% of HC cases and in 2–33% of CH series. In contrast, when PSH itself is the index disorder, accompanying TACs were infrequent, appearing in only 0–8% of PSH cases.

**Discussion:**

Based on both our clinical observations and the published data, PSH and migraine appear closely intertwined. Although the precise mechanisms remain speculative, the high degree of comorbidity underscores the possibility that PSH and migraine share overlapping pathophysiological pathways. Our findings lend support to the hypothesis that PSH may be facilitated by an underlying migraine biology. Clinically, our data may be an under-estimate as stabbing headache is generally not the presenting issue and may not have been well recorded. Future research aimed at elucidating molecular mechanisms could further clarify the nature of this relationship and pave the way for targeted therapies.

## Introduction

Primary stabbing headache (PSH) is defined in the International Classification of Headache Disorders, 3rd edition (ICHD-3) as a transient and localized stab, or series of stabs, of head pain that occur spontaneously, in the absence of an underlying lesion [1]. The entity has been described over the years under several appellations mirroring its phenotype. *Ophthalmodynia periodica* was the first label proposed by Lansche in 1964 to denote brief ocular jabs [2]. In 1979 Sjaastad and colleagues introduced the term “jabs-and-jolts syndrome”, to capture the sudden, electric-shock-like character of the attacks [3], while the colloquial term “ice-pick headache” soon entered the clinical vocabulary [4]. The 1988 first edition of the ICHD unified these variants under “idiopathic stabbing headache” [5], later renamed in the current classification as “primary stabbing headache”, to emphasize its nosological autonomy.

Epidemiological data indicate that PSH, while brief and easily overlooked, is not an uncommon phenomenon. A population-based study reported a lifetime prevalence of 35.2%, suggesting roughly one in three people have experienced these sharp head pains [6]. Overall, women seem to be affected more often than men – a pattern reminiscent of migraine – and most patients are diagnosed in adulthood, although cases across all ages have been documented [7–9]. Clinically, stabs may be localized in any scalp region, and laterality can change between attacks [10, 11]. Individual stabs last one to three seconds, and typically occur singly; however, volleys of several jabs in quick succession are also reported [10]. Importantly, attacks lack cranial autonomic features —features that help differentiate PSH from short-lasting unilateral neuralgiform headache attacks (SUNCT/SUNA) [12, 13]. Moreover, in contrast to trigeminal neuralgia, PSH is not induced, particularly by cutaneous triggers [14].

Beyond its intrinsic interest, PSH has a striking coexistence with other primary headache disorders, migraine being consistently reported as the most frequent comorbidity [15, 16], with rates of co-occurrence as high as 83% [17]. Notably, PSH is commonly reported to occur in trigeminal autonomic cephalalgias (TACs), such as cluster headache (CH) [18] and hemicrania continua (HC) [19]. This has led some authors to suggest that stabbing pains may represent an interictal expression of an underlying primary headache biology, proposing shared trigeminovascular hyperexcitability as a plausible link [16, 20].

Despite these observations, systematic data quantifying the strength and nature of the relationship between PSH and other primary headache disorders are sparse, and no consensus currently exists regarding the extent or clinical significance of this association. Prompted by a retrospective analysis of our PSH patients focusing on the co-occurrence with other primary headache disorders, we conducted a systematic review to explore this relationship.

## Methods

### Service evaluation

We conducted a service evaluation, which does not require ethics approval in the UK, as a single-centre retrospective cohort study at our tertiary headache clinic. Electronic medical records were searched for all encounters coded “primary stabbing headache” from 1 January 2018 through 31 March 2025. For every eligible patient we abstracted demographic information and documented the presence of other primary headache disorders, particularly migraine. In addition, data were collected on the reason for attendance, frequency and temporal characteristics of stabbing pains, co-localization with the underlying headache disorder, and whether treatments prescribed for the primary headache disorder had any effect on PSH. Given the retrospective design, not all variables were available for every patient, and missing data were coded accordingly. Because the study was descriptive in nature, we limited the analysis to summary statistics and did not perform formal hypothesis testing.

### Systematic review

**Fig. 1:**
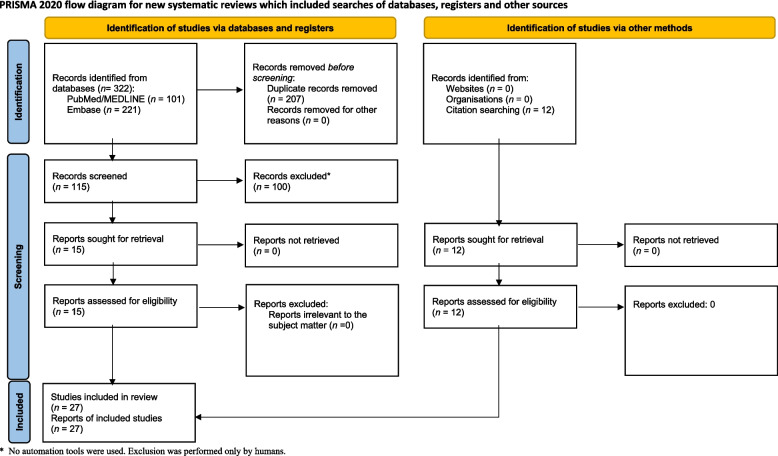
PRISMA Diagram

Parallel to the chart review, we carried out a systematic literature search following PRISMA guidelines [21]. PubMed/MEDLINE and Embase (via Scopus) were queried from database inception to 9 April 2025 using the terms “primary stabbing headache”, “idiopathic stabbing headache”, “jabs and jolts”, or “ice-pick headache” combined with “migraine”, “cluster headache” or “hemicrania continua”. Titles and abstracts were screened, full texts of potentially relevant studies were retrieved, and eligibility was assessed against predefined inclusion criteria: original research reporting on the relationship between PSH and other primary headache disorders. The sequential review steps, including the PRISMA flow diagram, are illustrated in Fig. 1. PJG provided senior oversight and critical revision of the manuscript throughout the study.

## Results

### Service evaluation

We retrospectively examined the charts of 68 patients with a definite diagnosis of primary stabbing headache. The cohort was predominantly female (82%), and the mean age at presentation was 46 ± 15 years. Concomitant migraine affected 61 patients (90%); of these, 44 (65%) met criteria for chronic migraine and 17 (25%) for episodic migraine, while aura was reported in 12 (18%). Outside of migraine, 7 patients (10%) had comorbid cluster headache and 5 (7%) fulfilled criteria for hemicrania continua. Only 2 patients (3%) had isolated primary stabbing headache without any additional primary headache disorder.

When examining the clinical context of the visits, information on the reason for attendance was available for 60 patients; among them, almost all (97%) attended primarily for their underlying headache disorder, whereas only a minority (3%) consulted due to PSH itself.

With regard to headache characteristics, data on frequency of stabs per day were available for 34 patients, with a mean of 7 ± 9 stabs/day. The number of days with stabbing headache per month was reported in 33 patients, with a mean of 17 ± 12 days. Information on co-localization of stabbing pain with the location of the underlying primary headache was present in 45 patients, and in this subset, 58% reported co-localization. Temporal association with migraine or cluster attacks could be assessed in 49 patients, among whom 69% reported that stabbing pains occurred predominantly during attacks, while 31% did not report this association.

Finally, information on the impact of primary headache disorder therapies on PSH was available for 14 patients. In this subset, 86% reported some improvement of stabbing pains when treated for their underlying headache disorder, whereas 14% noted no benefit. Results are summarized in Table 1. We note our clinical experience that PSH can be the primary complaint in children and adolescents, and can be very disabling.

**Table 1:** Demographic and baseline features

Variable	*n* (%)
Female sex	56 (82%)
Age, mean ± SD (years)	46 ± 15
Concomitant migraine	61 (90%)
• Chronic migraine	44 (65%)
• Episodic migraine	17 (25%)
Migraine with aura	12 (18%)
Cluster headache	7 (10%)
Hemicrania continua	5 (7%)
Pure PSH	2 (3%)

### Systematic review

From the systematic literature search, 27 articles were retrieved and included in the analysis. Overall, adult PSH cohorts consistently demonstrated high rates of migraine comorbidity, while migraine‑based samples showed a markedly elevated prevalence of stabbing headaches compared with non‑migraineurs. Paediatric PSH studies, though reporting lower concurrent migraine, revealed strong familial migraine patterns and associated episodic syndromes. Although with lower figures, PSH has been repeatedly described also in the context of TACs.

In adult PSH cohorts, Lansche’s seminal description reported that 66% of cases exhibited comorbid migraine [2]. Subsequent investigations confirmed this high prevalence, ranging from 25 to 83% [11, 17, 22–26] also sharing the same location when occurring concurrently [25]. In Atalar and colleagues’ population study, although only 32% met criteria for definite migraine, a migrainous biology was supported by high rates of accompanying symptoms—nausea in 68%, photophobia in 76% and phonophobia in 84% [27]. Finally, Seleker and colleagues demonstrated an 88% topographical concordance between stabbing and migraine headache in patients with both conditions. Further, they found that 79% of stabbing episodes occurred during migraine attacks, with a further 21% occurring immediately adjacent to them [28].

#### PSH in migraine

PSH has been observed at markedly elevated rates in migraine populations, with prevalence reported between 38 and 45% [29–32]. Case–control and cross‑sectional comparisons further underscored this association: Raskin and colleagues reported that PSH occurred in 42% of migraineurs versus 3% of controls, also having a strict temporal correlation in 69% of the cases [4], while Silva and colleagues reported that PSH was experienced by 44.7% of migraine sufferers compared to 10.3% of non‑migraine subjects [33].

#### PSH in TACs

In TACs cohorts, stabbing paroxysms characteristic of PSH have been frequently reported, with a prevalence of 36–41% in hemicrania continua series [19, 34] and in 2–33% of cluster headache series [18, 35–37]; additionally, Lance and Ekbom noted that these short-lasting jabs occurred during an ongoing cluster attack [18, 35]. By contrast, when PSH is the index condition, accompanying TACs are uncommon: HC appears in only 0–8% of adult PSH cohorts, while CH has not yet been reported [2, 11, 17, 22–27].

#### PSH in children

In paediatric PSH cohorts, comorbid migraine prevalence was lower, and ranged from 0 to 27% [38–42]. Family history of migraine was prominent across series, ranging from 35 to 78% [38–42]. Notably, Monte and colleagues reported that 72% of children in their cohort also experienced episodic syndromes such as cyclic vomiting and recurrent abdominal pain [42]. In these cohorts there was no report of comorbid CH or HC. The results are fully summarized in Table 2.

**Table 2:** Summary of studies investigating the relationship between PSH and other primary headache disorders

Group	Authors	Design	*N*	Main findings
Other primary headaches in Adult PSH Cohorts	Lansche [2]	Cohort	50	66% had comorbid migraine. No patients presented with CH or HC.
	Tugba et al. [17]	Cohort	31	Migraine present in 83% of PSH patients. No patients presented with CH or HC.
	Kim et al. [24]	Cohort	57	42% had concurrent migraine. No patients presented with CH or HC.
	Guerrero et al. [26]	Cohort	36	39% had migraine. No patients presented with CH, while 2 patients (6%) had concurrent HC.
	Pareja et al. [23]	Cohort	38	37% had migraine. No patients presented with CH or HC.
	Pedraza et al. [22]	Cohort	67	48% had migraine. No patients presented with CH, while 3 patients (4%) had concurrent HC.
	Cabral et al. [25]	Cohort	32	28% had migraine; half of comorbid cases shared identical location. No patients presented with CH or HC.
	Fuh et al. [11]	Cohort	80	25% reported migraine. No patients presented with CH or HC.
	Atalar et al. [27]	Population study	25	32% had definite migraine; notably, 68% had nausea, 76% photophobia, and 84% phonophobia accompanying stabs. Only 2 patients (8%) presented with TACs.
	Seleker et al. [28]	Cohort	43	88% had topographical overlap; 79% of stabs during, 21% around migraine attacks.
PSH in Migraine Cohorts	Raskin et al. [4]	Case–control	100 migraine patients vs 100 controls	PSH in 42% of migraineurs vs. 3% of controls; 69% of PSH episodes concurrent with migraine attacks.
	Silva et al. [33]	Population Study	1605	Idiopathic stabs in 44.7% of migraineurs vs. 10.3% of non-migraineurs.
	Piovesan et al. [32]	Cohort	233	40.4% of migraine patients presented idiopathic stabbing headache.
	Drummond & Lance [29]	Cohort	530	39% of migraine patients had PSH.
	Sjaastad et al. [30]	Population study	425	38% lifetime PSH among migraine patients.
	Sjaastad et al. [31]	Population study	178	45% lifetime PSH among migraine with aura patients.
Pediatric PSH Studies	Monte et al. [42]	Cohort	60	27% had migraine; 78% family history of migraine; 72% had episodic syndromes (e.g., cyclic vomiting). No patients presented with CH or HC.
	Soriani et al. [38]	Cohort	83	10% had migraine; 58% had family history of migraine. No patients presented with CH or HC.
	Raieli et al. [41]	Cohort	30	23% had coexisting migraine; 37% family history of migraine. No patients presented with CH or HC.
	Fusco et al. [40]	Cohort	23	No concurrent migraine, CH or HC. 35% had family history of migraine.
	Vieira et al. [39]	Cohort	20	No comorbid migraine, CH or HC. 45% had family history of migraine.
PSH in TACS Cohorts	Medina et al. [36]	Cohort	54	22% of CH patients had short-lasting jabs suggestive of PSH.
	Lance et al. [35]	Cohort	60	5% of CH patients had short-lasting jabs suggestive of PSH, concurrent with the attack.
	Ekbom [18]	Cohort	33	33% of CH patients experienced short-lasting jabs suggestive of PSH, concurrent with the attack.
	Wilbrink et al. [37]	Cohort	244	2% of CH patients had short-lasting jabs suggestive of PSH.
	Peres et al. [34]	Cohort	34	41% of HC patients had concurrent PSH.
	Cittadini & Goadsby [19]	Cohort	39	36% of HC patients had concurrent PSH.

## Discussion

Evidence drawn from our cohort and the systematic review supports an association between PSH and migraine. In our retrospective analysis of 68 consecutive PSH patients, we observed a co-occurrence with migraine in 90% of the cases. The literature search reinforces this link: adult PSH series consistently reported migraine, in up to 83% of cases, and migraine-based cohorts revealed that up to 45% of patients experience stabbing pains. In contrast, at a population level the lifetime prevalence was 38%. By contrast, paediatric PSH cohorts showed lower concurrent migraine (0–27%) with a high familial burden of migraine and a rich accompaniment of migraine-related episodic syndromes.

Regarding TACs, our cohort showed a relatively lower co-occurrence of cluster headache and hemicrania continua with PSH, Manifesting in 10% and 7% of cases, respectively. Likewise, from the literature search, CH and HC were present in 0–8% of PSH cases—figures that likely reflect the intrinsic rarity of these disorders. In CH series, PSH was reported in 2% to 33% of cases, whereas HC cohorts showed prevalences of 36–41%.

The pathophysiology of PSH is largely unknown, and animal models are lacking. Interestingly, PSH exhibits a distinctive sensitivity to indomethacin [43]. Although PSH is not formally classified among the indomethacin-responsive headache disorders, nearly two-thirds of patients achieve partial or complete relief with this drug [11, 23, 43], making it the mainstay of treatment [16, 44]. Indomethacin’s therapeutic value extends well beyond simple cyclo-oxygenase inhibition. The drug uniquely lowers cerebrospinal-fluid pressure [45], reduces cerebral blood flow [46], and interferes with nitric-oxide (NO) signalling [43, 47]. Notably, indomethacin not only blocks NO-induced vasodilatation [47] but, importantly, also suppresses the neuronal firing triggered by NO donors, unlike other NSAIDs [48]. This may be important since NO is involved in several migraine-related pathways. In the trigeminal ganglion, nitric-oxide synthase colocalises with calcitonin gene–related peptide (CGRP) [49], and experimental models show that NO can drive CGRP release [50]. Moreover, NO facilitates glutamate-mediated activation of NMDA receptors [51, 52], a mechanism increasingly recognised as pivotal in migraine [53]. Importantly, NO promotes central sensitisation in migraine, leading to a hyperexcitable state in peripheral and central pain circuits [54–56]. Within this sensitised milieu, spontaneous discharges from hyperexcitable neurons may surface as the brief, stabbing pains characteristic of PSH. Thus, these NO pathways may represent a link between PSH and other primary headache disorders, especially migraine, providing a pathophysiological substrate to explain their high rate of co-occurrence.

The paediatric data suggest that a migraine biology, although not yet sufficient to meet full diagnostic criteria for migraine, may nonetheless underlie or facilitate PSH in children. High rates of family migraine history (35–78%) and the frequent presence of migraine-correlated episodic syndromes suggest that the developing trigeminovascular system may already be primed toward hyperexcitability. Transient, focal network discharges in such a primed system could therefore manifest as stabbing pains before the brain matures into the full migraine phenotype later in life. Longitudinal studies would be needed to clarify this topic, and represent an intriguing area of investigation.

Cluster headache has been historically associated with short-lasting paroxysms named “cluster-tics”, due to the shared phenotype with trigeminal neuralgia. First described by Klimek [57], the patients have both typical cluster headache and separately clear trigeminal neuralgia, the latter responding to treatments, such as carbamazepine, which are not effective in cluster headache. Importantly, when tested, such patients’ stabs do not respond to indomethacin [58–60]. However, given the frequent absence of trigger factors, and localization within the first branch of trigeminal nerve, it has been suggested that these short-lasting jabs could be explained by PSH [9, 37]. These cohorts seem to mix the classic presentation of Klimek with a PSH presentation. Recently, a new definition of “cluster stabs” was proposed, following a study on 424 CH patients that found short-lasting jabs affecting 47% of cases, independently from TN [61]. The authors excluded patients who had trigeminal neuralgia from the analysis, thus excluding the classic Klimek phenotype. The authors described cluster stabs as short-lasting paroxysms, usually isolated to the first branch of the trigeminal nerve, triggerable only in 17% of cases, with possible, albeit non prevalent, autonomic manifestations, and suggested that cluster stabs should be considered part of the CH symptomatology [61]. It seems likely this represents PSH in cluster headache and the term “cluster stabs” is simply unnecessary. Lastly, it is worthwhile to consider epicrania fugax, another similar disorder characterized by dynamic short-lasting paroxysms that spread over the head [62], of stabbing or electric quality [63]. This is likewise frequently described occurring alongside migraine or cluster headache [64–68]. This might be best regarded as an endophenotype of PSH.

### Limitations

The retrospective nature of our cohort means that case ascertainment is dependent on clinical documentation: stabbing pains are rarely the primary complaint and may therefore be under-reported, leading to a potential under-estimate of true prevalence. In addition, the tertiary referral status of our center may have influenced the high proportion of chronic migraine. It should be noted that several clinical variables were not consistently available, a limitation that is inherent to the retrospective design. Finally, the hypothesis presented is speculative, as focused studies on pathophysiology of PSH are lacking, and therefore further work is needed to explore more precisely the interplay between PSH and other primary headache disorders.

## Conclusion

Based on our clinical observations and the collective published data, primary stabbing headache appears closely intertwined with other primary headache disorders, especially migraine. Although definitive mechanisms remain speculative, the consistently high comorbidity suggests that the two entities may share overlapping pathophysiological pathways, potentially involving nitric oxide. Our series may underestimate true prevalence, because stabbing pains are seldom the primary reason for consultation, except in childhood, and are easily overlooked in routine records. Future work should focus on delineating the molecular underpinnings of PSH, thereby clarifying its relationship to migraine and other primary headache disorders, guiding more targeted therapeutic strategies.

## Data Availability

The datasets used and/or analysed during the current study are available from the corresponding author on reasonable request.
